# The Effects of Exercise on the Physical Fitness of High and Moderate-Low Functioning Older Adult Women

**DOI:** 10.1155/2016/8309284

**Published:** 2016-07-12

**Authors:** R. Christopher Mason, Michael Horvat, Joe Nocera

**Affiliations:** ^1^Delaware State University, Dover, DE 19901, USA; ^2^University of Georgia, Athens, GA 30602, USA; ^3^Emory University, Atlanta, GA 30322, USA

## Abstract

*Introduction*. Understanding how exercise affects individuals with varying levels of functional ability will provide further insight into the role of exercise during the aging process. It will also aid in the development of exercise programs that are appropriate for a wider spectrum of older adults. Specifically it was the primary aim of this study to determine and compare the effects of 10 weeks of community-based exercise on the cardiovascular endurance, muscular strength, flexibility, and balance fitness components of older adult women with high and moderate-low levels of physical function.* Methods*. Participants were placed in either the high functioning (*n* = 13) or moderate/low functioning (*n* = 17) groups based on their level of physical functioning. Fitness components were measured by the Senior Fitness Test and physical function was determined by the Composite Physical Function scale.* Results*. The results of the 3 × 2 mixed ANOVA statistical analysis showed no significant interaction effect for time ⁎ group for any of the six subtests (chair stand, arm curls, 2-minute step, chair sit-and-reach, back scratch, and 6-foot up-and-go) of the SFT. However, the main effect of time was significant for all fitness components and the main effect of group was significant for all fitness components except lower extremity flexibility.* Discussion*. Community-based exercise programs offering a variety of exercise types to people with varying levels of functional ability can be useful in maintaining or improving fitness and independence. These programs may also be capable of improving the self-efficacy of lower functioning older adults toward performing daily tasks.

## 1. Introduction

Participation in exercise and regular physical activity can provide numerous physiological, cognitive, and psychological health benefits in the aging population [1]. Impairments in fitness components such as muscle strength and balance influence the development of disability. In addition to muscle strength and balance, aerobic endurance, agility, mobility, and flexibility have also been shown to be significant determinants of physical independence [2]. This is consistent with the notion that physical fitness factors greatly on the ability to successfully perform routine daily tasks. This includes functional tasks such as simple housework, lifting and carrying objects, negotiating steps, and walking far enough to shop and complete errands [3, 4]. Defined as the capacity of an individual to carry out the physical activities of daily living, physical function is an independent predictor of functional independence, disability, morbidity, and mortality [5]. Aging is often associated with declines in physical function affecting vital processes that are critical to independence, social engagement, and quality of life [6]. Older adults transitioning toward disability may no longer be able to appropriately utilize and execute movement patterns associated with activities of daily living (ADL) as a result of declining physical fitness. Programs of regular exercise that include cardiorespiratory, resistance, flexibility, and neuromotor exercise beyond activities of daily living to improve and maintain physical fitness and health are then essential for most adults [5]. Although mature nervous system function and motor skills are achieved in the earlier stages of life, changes in movement patterns also occur during adulthood and the latter stages of the lifespan [7]. Older adults need to practice, learn new, and relearn known motor skills as part of task training, recreational pursuits, or rehabilitation [8]. Therefore, opportunities for exercise that foster fitness, efficient functional movement skills, and self-efficacy toward performing daily tasks are needed to impede the progression of the disablement process among older adults. As such, community-based modes of exercise aimed to equip older adults with neuromotor (balance, coordination, and agility), physical (aerobic endurance, muscle strength, and flexibility), and functional components of fitness necessary for daily life should be explored and developed [6].

As the population of older adults is continuing to increase and become more diverse, it will be important to recognize individual differences in level of functioning [9]. While aging is an inevitable process, it is an individual experience that has considerable variability. Older adults of the same chronological age may differ in physiological age and function due to differences in genetic makeup, lifestyle choices, cognitive ability, and several other variables. Exercise programs such as the one in this study should be designed to accommodate the variable capabilities and functional levels of older adult women. Additionally, they must be motivating enough to ensure active participation at an enjoyable level and increase the frequency of physical activity in a social, interactive setting. While this is true it is often the case that opportunities for exercise in community-based settings are not accommodating to individuals of all levels of functioning. Instruction of proper movement technique and motivational factors that promote adherence are often lacking. Exercise classes at community centers and facilities for adults will most likely contain individuals with varied levels of disability, as this is the nature of the older adult population. For example, a step aerobics class at the local senior center may have a participant who is highly functional and does not suffer from any level of disability exercising next to someone who is of the same age but has low cardiovascular endurance and poor balance. The effect and benefit of exercise on both types of individuals in this kind of environment are largely unknown. Therefore, the primary purpose of this study was to observe the exercise habits of older adult women with varying levels of physical function to determine any differential effects of exercise on their physical fitness.

## 2. Methods

### 2.1. Participants

A convenience sample of 30 women (*n* = 30, *m*_age_ = 69 years) recruited from the YWCO in Athens, Georgia, completed participation in this study. Participant demographics can be found in Table 1. Initially 37 women agreed to participate. However, seven participants were forced to withdraw for various reasons including hospitalization due to sickness, moving away, and summer travel. Participants were recruited in person while they attended exercise classes at the YWCO and also by posting flyers and sign-up sheets at the facility. All potential participants completed a medical history questionnaire to screen for conditions that may have inhibited safe exercise participation. Exclusion criteria included history of stroke, heart attack, osteoarthritis, neurological disease, mental illness, and fracture or joint replacement within the last six months. Individuals reporting one or more of such conditions were excluded from participation in this study. Additionally, the participant medical history form provided demographic information related to age, sex, height, weight, marital status, employment status, and dwelling status. Participants were also asked to report an average amount of time per week they routinely spent involved in planned exercise.

Two groups of participants were formed according to their physical ability to live independently as determined by the Composite Physical Function (CPF) scale. The CPF is a self-report 12-item scale capable of assessing physical function across a wide range of activities from basic ADLs such as bathing and dressing to instrumental ADLs including gardening and shopping [3, 4]. Each item can be scored from “0” to “2” (0 = cannot do; 1 = can do with help; 2 = can do without help) based on the participants perceived ability to perform the task in question. The CPF scale can be used to categorize individuals as “high functioning,” “moderate functioning,” or “low functioning” and at risk for loss of independence. High functioning individuals are those who indicate that they can perform all 12 items on their own without assistance, thus receiving a perfect score of 24. Both the definition of moderate functioning and its interpretation are adjusted for age. It is important that younger age groups have more stringent criteria than older age groups for being assessed as moderate functioning as age-related declines in physical capacity after the age of 60 are commonly reported to decline at least 10%–15% per decade [10]. Whereas a score of 14 (ability to perform a minimum of seven CPF activities without assistance) is required for a rating of moderate of those aged 90 years and older, higher scores of 20, 18, and 16, respectively, are needed in order for those in their 60s, 70s, and 80s to be rated as moderate functioning [3, 4]. Using this age-adjusted scoring the participants were placed in either the high functioning (*n* = 13) or moderate/low functioning (*n* = 17) groups.

### 2.2. Procedure

All participants were required to read and sign the University of Georgia Institutional Review Board consent form prior to commencing any screening, testing, or exercising. As part of a Senior Fitness Initiative, each participant from both groups was encouraged to continue their normal routine of attending group exercise classes at the YWCO and also to exercise on their own at home for a 10-week period. Weekly exercise logs were used throughout the study as a method of self-reporting to record and compare the exercise habits of participants from each group with varying levels of functional independence. The participants were instructed to use the weekly exercise log to record each bout of planned exercise in which they participated during the 10-week study. The Senior Fitness Test (SFT) [3, 4] was used to assess the overall fitness of both groups of participants at baseline, after five weeks, and again after 10 weeks of exercising. The SFT is commonly used to assess physical fitness in older adults, as it represents an easy-to-use field test battery that allows for the assessment of physical fitness components vital to maintaining independent functioning [11].

### 2.3. Exercise and Fitness Assessments

#### 2.3.1. Weekly Exercise Log

A weekly exercise log was given to each participant of them to record the type and duration of each bout of exercise. Participants only received credit for planned exercise and not for other forms of physical activity such as housework, grocery shopping, or doing laundry. The weekly exercise logs were used as a method of self-reporting to record and compare the exercise habits of participants from each group with varying levels of functional independence. Participants received reminders to continue tracking their exercise in-person and by email on a weekly basis. Weekly exercise logs were submitted for review at the midpoint and at the end of the 10-week exercise period. The number of weekly minutes of exercise performed by the high functioning and moderate/low functioning groups was then tallied and recorded at these time points. The average number of minutes exercised was then calculated for each group after five weeks and 10 weeks of exercise.

#### 2.3.2. Senior Fitness Test

The Senior Fitness Test consists of seven items, including one alternate test for measuring aerobic endurance. The SFT items are as follows: (1) 30-second chair stand; (2) arm curl; (3) chair sit-and-reach; (4) back scratch; (5) 6-minute walk; (6) 2-minute step; and (7) 8-foot up-and-go. The purpose of the 30-second chair stand is to measure lower extremity strength, which is necessary for tasks such as transferring from a chair, walking, and climbing stairs. The total number of complete stands able to be completed in 30 seconds was recorded. The arm curl component of the SFT measures upper extremity strength, which is needed to perform household activities such as lifting and carrying groceries, suitcases, or grandchildren. The total number of bicep curls completed with correct form with the dominant arm while holding a five-pound weight in 30 seconds was recorded. The chair sit-and-reach test will be administered to assess lower body flexibility, which is important for good posture and normal gait patterns. Lower body flexibility also contributes to performing mobility tasks such as getting in and out of a car or bed. From a sitting position at the end of a chair, with one leg extended and hands reaching toward toes, the number of inches (+ or −) between extended fingers and tip of toe was measured during the chair sit-and-reach test. Upper body flexibility was measured by the back scratch test. Shoulder flexibility is vital for tasks such as combing one's hair, putting on a coat, and reaching for a seat belt. During this test, the number of inches between middle fingers (+ and −) was measured while reaching over the shoulder with arm and up the middle of the back with the other arm. The 2-minute step test was chosen for the assessment of aerobic endurance because space and time limited the use of the 6-minute walk. Aerobic endurance is important for walking distances, climbing stairs, and other activities such as shopping or sightseeing. As such, cardiovascular fitness was measured with the 2-minute step test which entails recording the number of full steps completed in two minutes while raising each knee to a point midway between the patella and iliac crest. The recorded score was equal to the number of times the right knee reaches the required height. The final component of the SFT is the 8-foot up-and-go test and is intended to assess agility and dynamic balance. This fitness component is essential for quick maneuvering which is required for activities such as rushing to answer a telephone or to use the restroom. The 8-foot up-and-go test requires an individual to rise from a chair, walk forward 8 feet, change direction, and return to their seated position in the chair. The fastest time from two trials was recorded to the nearest hundredth of a second. Each test item of the SFT has accompanying performance standards for men and women ages 60 to 94-plus based on a national study of more than 7,000 Americans [3, 4]. Additionally, the SFT provides threshold values on each test item that help to identify if an older adult is at risk for mobility loss.

#### 2.3.3. Exercise Protocol

For a 10-week period participants were monitored while performing their normal exercise routines within the Athens, GA, community. The majority of this exercise took place at the Athens YWCO with the remaining exercise taking place at home or in other settings. While at the YWCO, participants attended a variety of exercise classes designed for older adults. The classes were either 30 or 60 minutes in duration and collectively emphasized all components of physical fitness. There was no limit placed on the participants in terms of type or amount of classes they were allowed to attend in each week. It was common for the women in both groups to exercise together in the same classes and attend different classes on an individual basis. The exercise classes offered at the YWCO during the time of the study focused on functional movements and included Group Strength Training, Pilates, Silver Sneakers, and Step and Sculpt. After baseline testing the participants were educated on how to execute proper posture during exercise and while performing daily activities. The researchers collaborated with the YWCO instructors to ensure that proper execution of functional movements was emphasized during class time. Instructors were also asked to associate the movements being taught to activities of daily living. For example, when performing arm strengthening exercises instructors were asked to relate these movements to functional tasks such as lifting and carrying items similar to laundry baskets, grocery bags, or grandchildren. The type and duration of class participation and other exercise activities were recorded on the weekly exercise log.

### 2.4. Statistical Analysis

#### 2.4.1. Senior Fitness Test

The Senior Fitness Test was given to each group before, at midpoint, and following 10 weeks of community-based exercise. The Senior Fitness Test (SFT) measures the components of fitness with a chair stand test, arm curl test, 2-minute step test, chair sit-and-reach test, back scratch test, and 8-foot up-and-go test. Each test is scored separately as there is no composite score for the SFT. As such, a separate mixed 3 (time) × 2 (group) ANOVA, with time as the within-subjects factor and group based on level of physical function as the between-subjects factor, was run for each subtest of the SFT. Analyses were conducted using SPSS 22 software (SPSS IBM, New York, USA). The *p* = 0.05 rejection level was used in all analyses.

#### 2.4.2. Weekly Self-Reported Exercise

The number of weekly minutes of exercise performed by the high functioning and moderate/low functioning groups was tallied and recorded at the midpoint and after 10 weeks of exercise. The estimation of time spent exercising reported on the health and medical history questionnaire before the study was used as the preexercise value for self-report exercise.

## 3. Results

### 3.1. Senior Fitness Test

**Figure 1:**
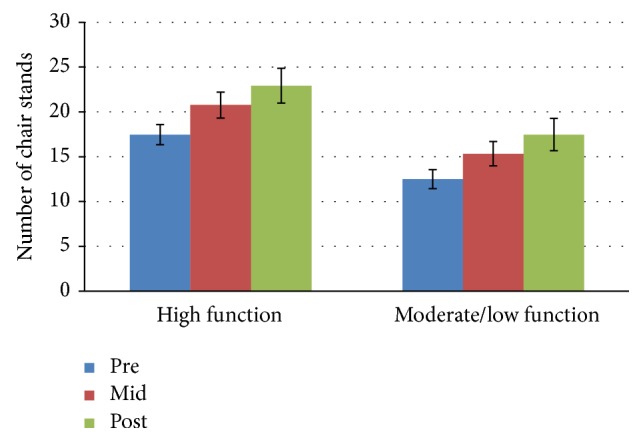
Chair Stand Test mean scores by group and time.

**Figure 2 fig2:**
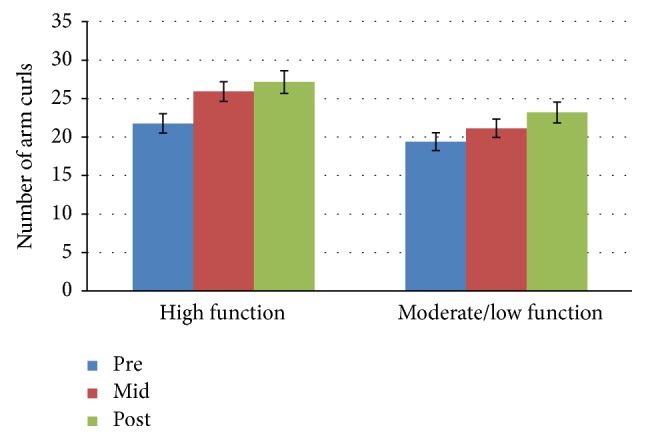
Arm curl test mean scores by group and time.

**Figure 3 fig3:**
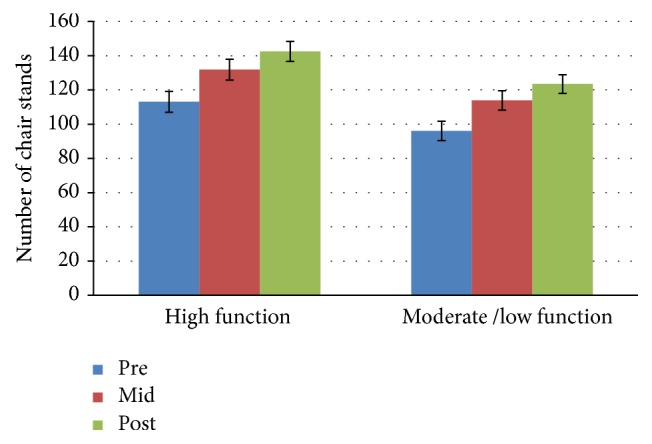
Two-minute step test mean scores by group and time.

**Figure 4 fig4:**
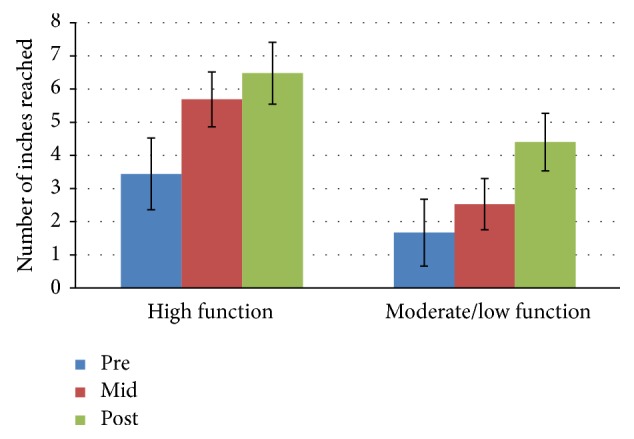
Chair sit-and-reach test mean scores by group and time.

**Figure 5 fig5:**
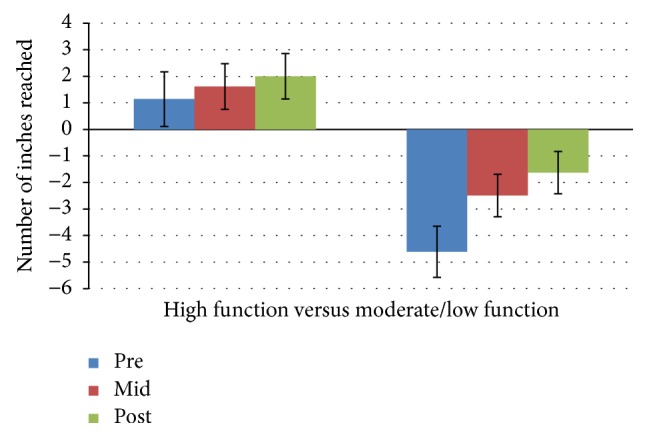
Back scratch test mean scores by group and time.

**Figure 6:**
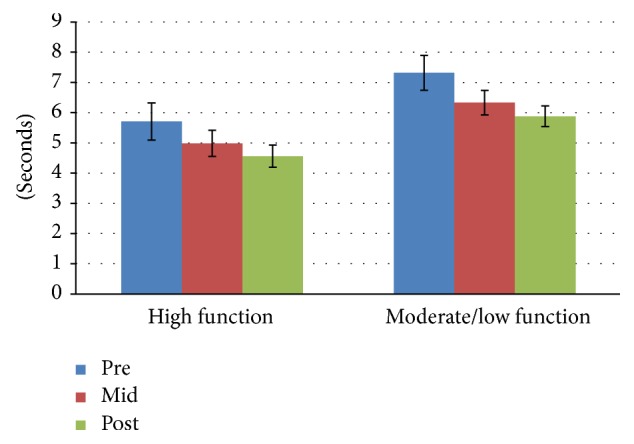
Eight-foot up-and-go test mean scores by group and time.

The results of the 3 × 2 mixed ANOVA statistical analysis showed no significant interaction effect for time*∗*group for any of the six subtests (chair stand, arm curls, 2-minute step, chair sit-and-reach, back scratch, and 6-foot up-and-go) of the SFT. However, the main effect of time was significant for all fitness components and the main effect of group was significant for all fitness components with the exception of lower extremity flexibility (chair sit-and-reach test). Means and standard deviation for each of the six subtests scores for both groups are presented in Figures 1 [Fig fig2] [Fig fig3] [Fig fig4] [Fig fig5]–6. The results for each SFT subtest are reported below.

### 3.2. Chair Stand Test

Statistical analysis showed no significant interaction effect for time *∗* group: *F*(2,52) = 0.089, *p* = 0.915, and *n* ^2^ = 0.003 for number of chair stands at any of the three time points. The main effect of time showed a statistically significant difference in chair stands at the 3 time points: *F*(2,52) = 26.983, *p* < 0.0005, and partial *n* ^2^ = 0.509. The main effect of group showed a statistically significant difference in chair stands between physical function groups: *F*(1,26) = 7.387, *p* = 0.012, and partial *n* ^2^ = 0.221. The mean scores for the high function group for pre-, mid-, and posttests were 17.5, 20.8, and 22.9, respectively. Percent change from baseline to midpoint was 18.8%. The high function group mean score for chair stands also increased from midpoint to posttest with a percent change of 10.1%. The moderate/low function group showed larger percent changes between time points. Mean scores for the moderate/low function group increased from 12.5 to 15.3 yielding a percent change of 22.4%. Percent change between midpoint and posttest was 14.4% with mean scores increasing from 15.3 to 17.5.

### 3.3. Arm Curls Test

Mean scores for the high function group increased over time and were 21.8, 25.9, and 27.15 for pre-, mid-, and posttests, respectively. Percent change between baseline and midpoint was 18.8% and was 4.8% between midpoint and posttest. The moderate/low function group mean scores at pre-, mid-, and posttests were 19.4, 21.3, and 23.2. Baseline to midpoint percent change was calculated to be 9.8% and midpoint to posttest percent change was 8.9%. Statistical analysis showed no significant interaction effect for time *∗* group: *F*(2,52) = 0.743, *p* = 0.481, and partial *n* ^2^ = 0.028 at any of the data collection time points. The main effect of time showed a statistically significant difference in arm curls at the three time points: *F*(2,52) = 10.636, *p* < 0.0005, and partial *n* ^2^ = 0.290. The main effect of group showed a statistically significant difference in arm curls between physical function groups: *F*(1,26) = 6.969, *p* = 0.014, and partial *n* ^2^ = 0.211.

### 3.4. Two-Minute Step Test

The high function group averaged 113, 131.9, and 142.54 steps at the three data collection time points. The percent change between baseline and midpoint was 16.7% and was calculated to be 8.1% between midpoint and posttest. The moderate/low function group mean scores increased by 18.5% between baseline and midpoint and also increased between midpoint and posttest with a percent change of 8.4%. There was no significant interaction effect for time *∗* group for the 2-minute step test: *F*(2,50) = 0.032, *p* = 0.853, and partial *n* ^2^ = 0.001 at any of the three data collection time points. The main effect of time showed a significant difference in steps at the 3 time points: *F*(2,50) = 14.08, *p* < 0.0005, and partial *n* ^2^ = 0.360. The main effect of group showed a significant difference in steps between physical function groups: *F*(1,26) = 10.71, *p* = 0.003, and partial *n* ^2^ = 0.292.

### 3.5. Chair Sit-and-Reach Test

There was no significant interaction effect for time *∗* group for the chair sit-and-reach test: *F*(2,52) = 0.967, *p* = 0.387, and partial *n* ^2^ = 0.036. The main effect of time showed a significant difference in inches reached at the 3 time points: *F*(2,52) = 15.235, *p* < 0.0005, and partial *n* ^2^ = 0.369. However, the main effect of group did not show a significant difference in inches reached between physical function groups: *F*(1,25) = 4.09, *p* = 0.054, and partial *n* ^2^ = 0.136. The high function group increased their mean scores by 67.6% and 14% between baseline and midpoint and between midpoint and posttest, respectively. The moderate/low function group increased their mean scores by 47% between baseline and midpoint and also increased mean scores by 76% between midpoint and posttest.

### 3.6. Back Scratch Test

There was no significant interaction effect for time *∗* group for inches reached on the back scratch test: *F*(2,52) = 1.1, *p* = 0.341, and partial *n* ^2^ = 0.115 at any of the three data collection time points. The main effect of time showed a significant difference in inches reached at the 3 time points: *F*(2,50) = 3.321, *p* = 0.044, and partial *n* ^2^ = 0.117. The main effect of group showed a significant difference in inches reached during the back scratch test between physical function groups: *F*(1,26) = 24.374,  *p* < 0.0005, and partial *n* ^2^ = 0.484. The high function group increased their mean scores by 45.7% and 25% between baseline and midpoint and between midpoint and posttest, respectively. The moderate/low function group increased mean scores by 47.8% between baseline and midpoint and also increased mean scores by 36% between midpoint and posttest.

### 3.7. Eight-Foot Up-and-Go Test

There was a 12.6% percent change for the high function group between pre- and posttest. The percent change for the same group between midpoint and posttest was 7.6%. The moderate to low function group improved by 13.7% and 6.3%, respectively, during the two periods between data collection time points. There was no significant interaction effect between time *∗* group for the 8-foot up-and-go test: *F*(2,52) = 0.06, *p* = 0.942, and partial *n* ^2^ = 0.002. The main effect of time showed a significant difference between midpoint and postexercise tests: *F*(2,52) = 4.099, *p* = 0.022, and partial *n* ^2^ = 0.136. The main effect of group showed a significant difference in up-and-go scores between physical function groups: *F*(1,26) = 13.071, *p* = 0.001, and partial *n* ^2^ = 0.335.

## 4. Discussion

The benefits of exercise for older adults have been established in the research literature and are well known. There is evidence that habitual exercise can minimize the physiological effects of an otherwise sedentary lifestyle and prolong active life expectancy [12]. The heterogeneity observed among older adults and their level of physical function has important implications for research and clinical practice. This notion supports the concept that tailoring exercise programs and interventions to specific deficits and the current state of physical functioning is an important consideration. Research has left little doubt that exercise interventions for older adults need to consider the individual functional needs of participants. As such, the success of aerobic and resistance training for obese men and women [13], balance training for individuals at risk for falls, and water exercise for those with osteoarthritis [14] have all been shown to be effective for older adults with these specific physical function deficits. Despite the plethora of data showing an overall benefit of exercise training for improving, or maintaining functional ability in older adults, there is likely to be large individual variability in functional responses to exercise. Attention to individual differences and identification of factors that influence efficacy of exercise as a therapy for aging-related loss of physical function have important clinical significance. Moreover, the specific amount of exercise necessary to elicit maximal improvements in physical outcomes may differ between types of individuals. Exercise training studies that show efficacy for improving functional ability often report main effects or mean group differences without expressing the extent of variability for these tasks. Therefore, it was the primary purpose of this study to observe the exercise habits of older adult women with different levels of physical function abilities, to determine any differential effects of exercise on their physical fitness.

The specific aim of the study was to determine and compare the effects of 10 weeks of community-based exercise on the cardiovascular endurance, muscular strength, flexibility, and balance fitness components of older adult women with high and moderate-low levels of physical function. The results of the statistical analysis indicate that the impact of time (10 weeks of exercise) on the fitness-related outcome measures did not differ based on level of physical function at any time during the study. In other words, older adult women from this sample were able to improve fitness over time regardless of their initial level of physical function as indicated by their initial score on the Composite Physical Function scale and group assignment. Over the course of 10 weeks the women from both groups improved their SFT scores related to leg strength, arm strength, cardiovascular endurance, leg flexibility, arm flexibility, and mobility and dynamic balance. This finding is consistent with the hypothesis that all participants would increase their scores on all SFT subtests over time. The exercise classes offered at the YWCO provided the participants with a variety of options in terms of which fitness components to train. Many of the exercise classes such as Pilates and Silver Sneakers emphasized multiple fitness components making them highly beneficial to the overall fitness of the participants. By offering programs of regular exercise that include cardiorespiratory, resistance, flexibility, and neuromotor exercise beyond activities of daily living, the Athens YWCO is equipped to improve and maintain the fitness and health of its members [5]. Additionally, the implementation of the Senior Fitness Initiative at the YWCO greatly influenced the participants in terms of their morale and enthusiasm toward exercise. They were highly engaged and eager to learn more about their individual level of fitness.

While the high functioning group consistently had significantly better SFT scores as compared to their counterparts, they did not improve at a greater rate or more significantly over time as compared to the moderate/low functioning group. However, it should be noted that the percent change during the second half of the study between midpoint and posttests was higher for the moderate/low functioning group for all six subtests of the Senior Fitness Test. This implies that the group with lower amounts of functional ability continued to improve and were more resistant to the plateau effect shown by the high function group. The lower functioning individuals seemed to be inspired by their gains and were highly motivated to keep pace with the other women in the study. This finding suggests that there may be some benefit to integrating community-based exercise classes with people of various functional skill sets when resources or logistics are not conducive to separating them. This should only be done with qualified instructors who are able to offer modifiable exercise opportunities to the variable older adult population.

As it was also an aim of this study to compare the exercise habits of older adult women functioning at different levels of independence, self-report weekly exercise logs were used to track the time spent exercising of each participant. Prior to the 10-week exercise period, both groups were asked to estimate the amount of time they spent engaged in exercise on a weekly basis. An interesting finding was that the actual number of recorded minutes during the study (at midpoint and after study) was lower than the estimated averages reported by both groups prior to commencing the 10-week exercise period. Perhaps this can be explained by social desirability, which can lead to overreporting of physical activity [15]. This common limitation of self-report instruments appears to have held true for the weekly exercise logs and this sample of older adult women. Despite this limitation, there were some benefits of utilizing the weekly exercise logs to self-report exercise activity. The participants often reported the feeling of being held accountable for their exercise activity because they were forced to write it down and track it over 10 weeks. The weekly exercise logs seemed to produce a sense of pride among the participants and may have motivated them to be more physically active. Initiating exercise programs and adhering to them is often problematic for older adults [16]. Using weekly exercise logs may help to alleviate this problem among older adults. Also, all participants consistently reported performing exercise activity at home and in other settings outside of the YWCO. This indicates that Senior Fitness Initiatives such as the one implemented during this study may be helpful in improving the overall exercise habits of older adults. These findings suggest that, although sometimes limited in effectiveness, self-report instruments to track the exercise habits of older adults in a community-based setting may be useful when more scientific and reliable options are not available. It is possible that these instruments may have cognitive and physical benefits for older adults. Their use requires the highly complex task of recall and may motivate individuals to be more active by holding them accountable for the amount of time they choose to be engaged in exercise. Additionally, study findings suggest that participation in fitness and exercise initiatives in a community-based setting can improve the perceived ability of older adults to function independently.

In conclusion, this study provides a few noteworthy findings. First, community-based exercise programs offering a variety of exercise types to people with varying levels of functional ability have both physical and psychosocial benefits. These programs can be useful in maintaining and improving fitness and independence. Second, these programs may also be capable of improving the self-efficacy of lower functioning older adults toward performing daily tasks. Finally, self-report instruments such as activity logs may be useful to track and gain an understanding of the exercise habits of older adults. This study was limited due to the nature of the community from which the participants were recruited. The YWCO members were highly educated and most already had an established history of physical activity and exercise. Additionally they were extremely competitive and eager to improve. Future research should build upon using multimodal exercise regimes focusing on proper format, periodic assessment and feedback, and encouragement of home-based activities. More importantly exercise programs should teach older adults to move correctly while generalizing movements to activities commonly encountered by the population.

## Figures and Tables

**Table 1:** Demographics and clinical features of participants by group.

	High function	Moderate/low function
Number of women	*n* = 13	*n* = 17
	Mean (SD)	Mean (SD)
Age (years)	67.2 (5.7)	70.2 (5.1)
CPF score	24	20.4 (3.7)
Exercise (min/week)	803 (715)	526 (377)
